# Skin Grafting

**Published:** 1899-12-02

**Authors:** 


### SKIN GRAFTING.

Dr. T. H. Killoch, of the Middlesex Hospital, describes a new method of skin grafting in which, while the centre of the graft contains the whole thickness of the skin, this is surrounded by flaps consisting of epithelium only, as in the Thiersch method, the object being to give the new scar a greater degree of suppleness and expansibility than is obtainable where a Thiersch graft is applied direct on to surfaces which have been somewhat deeply excavated either by operation or disease—as, for example, about the face and neck and at the flexures of the joints. The method seems likely to be of much service in such cases. Dr. Killoch insists on a point, inattention to which has, we think, often been the cause of disappointment in the use of Thiersch grafting, namely, the avoidance of antiseptics at the time of the operation. He says, "It has been shown by experiment that although the vitality of epithelial grafts is not impaired by keeping for a considerable time, or by moderate degrees of heat and cold, they are peculiarly susceptible to the action of chemical antiseptics; these surfaces, then—those from which the grafts are to be obtained—after having been rendered aseptic should be treated for at least twenty-four hours' operation with some aseptic dressing, such as sterilised gauze or lint wrung out of plain boiled water, similar precautions being taken with regard to the instruments used at the operation and the dressings applied at the time, so that no chemical antiseptics may come in contact with the grafts until they become adherent in the new position."

